# 2,3,4-Trihy­droxy­benzoic acid 0.25-hydrate

**DOI:** 10.1107/S160053681200709X

**Published:** 2012-02-24

**Authors:** Jin-Hang Li, Fu-Yue Dong, Fang Cai, Xiao-Feng Yuan, Ren-Wang Jiang

**Affiliations:** a Guangdong Province Key Laboratory of Pharmacodynamic Constituents of, Traditional Chinese Medicine and New Drugs Research, Institute of Traditional Chinese Medicine and Natural Products, Jinan University, Guangzhou 510632, People’s Republic of China

## Abstract

The asymmetric unit of the title compound, C_7_H_6_O_5_·0.25H_2_O, contains two mol­ecules of 2,3,4-trihy­droxy­benzoic acid, with similar conformations, and one water mol­ecule which lies on a twofold rotation axis. Both acid mol­ecules are essentially planar [maximum r.m.s deviations = 0.0324 (2) and 0.0542 (3) Å for the two acid molecules]. The mol­ecular conformations are stabilized by intra­molecular O(phenol)—H⋯O(carbox­yl/phenol) inter­actions. A cyclic inter­molecular association is formed between the two acid and one water mol­ecule [graph set *R*_3_^3^(12)] involving O—H⋯O hydrogen bonds. The two acid mol­ecules are further linked through a cyclic *R*_2_^2^(8) carb­oxy­lic acid hydrogen-bonding association, which together with inter­molecular O—H⋯O hydrogen-bonding inter­actions involving the phenol groups and the water mol­ecule, and weak π–π inter­actions [minimum ring centroid separation = 3.731 (3) Å], give a three-dimensional network.

## Related literature

For the natural distribution of 2,3,4-trihy­droxy­benzoic acid, see: Zhai *et al.* (2010 ▶); Xu & Chang (2010 ▶). For its anti­oxidant and anti­bacterial activities, see: Kodama *et al.* (2007 ▶); Friedman *et al.* (2003 ▶). For the inhibition of xanthine oxidase, see: Chang *et al.* (1995 ▶). For the crystal structure of the dihydrate pseudopolymorph, see: Prior & Sharp (2010 ▶). For π–π inter­actions in gallic acid pyridine monosolvate and in natural flavonoids, see: Dong *et al.* (2011 ▶); Jiang *et al.* (2009 ▶, 2002 ▶). For graph-set analysis, see: Etter *et al.* (1990 ▶); Bernstein *et al.* (1995 ▶).
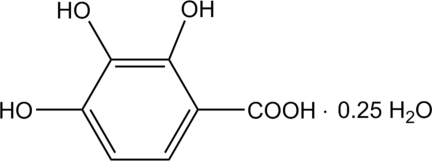


## Experimental

### 

#### Crystal data

C_7_H_6_O_5_·0.25H_2_O
*M* *_r_* = 174.62
Orthorhombic, 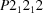

*a* = 11.8364 (12) Å
*b* = 32.598 (3) Å
*c* = 3.7306 (4) Å
*V* = 1439.4 (3) Å^3^
*Z* = 8
Mo *K*α radiation
μ = 0.14 mm^−1^
*T* = 291 K
0.42 × 0.28 × 0.20 mm

#### Data collection

Bruker SMART 1000 CCD diffractometer
Absorption correction: multi-scan (*SADABS*; Sheldrick, 2004 ▶) *T*_min_ = 0.874, *T*_max_ = 1.000
7863 measured reflections
2552 independent reflections
2010 reflections with *I* > 2σ(*I*)
*R*_int_ = 0.060

#### Refinement

*R*[*F* ^2^ > 2σ(*F* ^2^)] = 0.036
*wR*(*F* ^2^) = 0.090
*S* = 0.97
2552 reflections
222 parameters
H-atom parameters constrained
Δρ_max_ = 0.17 e Å^−3^
Δρ_min_ = −0.15 e Å^−3^

### 

Data collection: *SMART* (Bruker, 1998 ▶); cell refinement: *SAINT* (Bruker, 1998 ▶); data reduction: *SAINT*; program(s) used to solve structure: *SHELXS97* (Sheldrick, 2008 ▶); program(s) used to refine structure: *SHELXL97* (Sheldrick, 2008 ▶); molecular graphics: *XP* (Siemens, 1998 ▶); software used to prepare material for publication: *SHELXTL* (Sheldrick, 2008 ▶).

## Supplementary Material

Crystal structure: contains datablock(s) I, global. DOI: 10.1107/S160053681200709X/zs2181sup1.cif

Structure factors: contains datablock(s) I. DOI: 10.1107/S160053681200709X/zs2181Isup2.hkl

Supplementary material file. DOI: 10.1107/S160053681200709X/zs2181Isup3.cml

Additional supplementary materials:  crystallographic information; 3D view; checkCIF report

## Figures and Tables

**Table 1:** Hydrogen-bond geometry (Å, °)

*D*—H⋯*A*	*D*—H	H⋯*A*	*D*⋯*A*	*D*—H⋯*A*
O1*W*—H1*WA*⋯O2	0.82	2.19	2.963 (2)	156
O1—H1*A*⋯O4	0.82	1.88	2.587 (3)	143
O2—H2*A*⋯O2′^i^	0.82	2.05	2.839 (4)	161
O3—H3*A*⋯O3′^ii^	0.82	2.06	2.828 (3)	155
O5—H5*B*⋯O4′^iii^	0.82	1.88	2.679 (4)	165
O1′—H1′*A*⋯O4′	0.82	1.87	2.588 (3)	145
O2′—H2′*A*⋯O1	0.82	1.95	2.729 (4)	159
O3′—H3′*A*⋯O1*W*	0.82	2.06	2.841 (3)	158
O5′—H5′*B*⋯O4^iv^	0.82	1.85	2.659 (4)	171

Symmetry codes: (i) 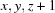; (ii) 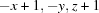; (iii) 
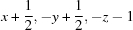
; (iv) 
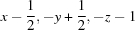
.

**Fig. 1.**
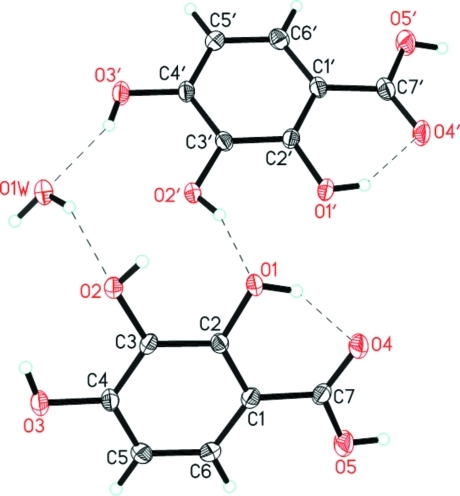
An ORTEP plot of the asymmetric unit of the title compound with atoms shown as 30% probability ellipsoids. The water molecule lies on a crystallographic twofold rotation axis and hydrogen bonds are shown as dashed lines.

**Fig. 2.**
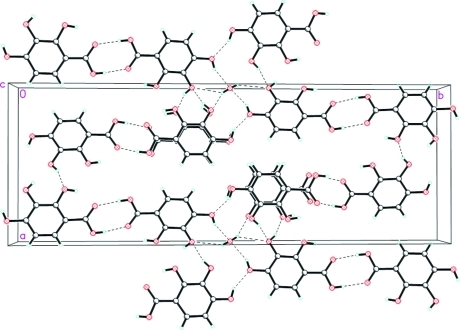
Packing diagram viewed down the c-axis showing hydrogen bonds as dashed lines.

## supplementary crystallographic information

### Comment

2,3,4-Trihydroxybenzoic acid is a polyphenolic acid which has been isolated from *Pachysandra terminalis* (Zhai *et al.*, 2010) and the lentil (Xu & Chang, 2010). It has been found to show antioxidant (Kodama *et al.*, 2007) and antibacterial activities (Friedman *et al.*, 2003) and also to inhibit the xanthine oxidase enzyme (Chang *et al.*, 1995). This compound contains two of the most common functional groups in natural products: the carboxylic acid and the phenolic groups. The dihydrate pseudopolymorph of the acid with a space group *P*-1 has previusly been reported (Prior & Sharp, 2010). In this study, we report the structure of the second pseudopolymorph, the partial hydrate C_7_H_6_O_5_ . 0.25H_2_O.

The asymmetric unit of the title compound contains two molecules of 2,3,4-trihydroxybenzoic acid [(1) and (2)] with similar conformations, and one water molecule of solvation which lies on a crystallographic twofold rotation axis (Fig. 1). Both acid molecules are essentially planar [maximum r.m.s deviation 0.0324 (2) and 0.0542 (3) Å for the two acid molecules], with a dihedral angle of 49.4 (3)° between the planes. The molecular conformation of the acid molecules is stabilized by a number of intramolecular phenolic O—H···O interactions (Table 1).

The water molecule and the acid molecules are linked through O—H···O hydrogen bonds (Table 1) with both acting as donors and acceptors, giving a cyclic association [graph set R^3^_3_(12) (Etter *et al.*, 1990; Bernstein *et al.*, 1995)]. The 2,3,4-trihydroxybenzoic acid molecules form head-to-head pairs through intermolecular hydrogen bonds [graph set R^2^_2_(8). The molecular pairs are further extended into chains through hydrogen bond O2–H···O2'^i^ , while adjacent chains are connected *via* hydrogen bond O3–H···O3'^ii^ into a three-dimensional network (Fig. 2) [for symmetry codes (i) and (ii), see Table 1]. It is noteworthy that aromatic π–π associations also play an important role in the molecular packing. Adjacent 2,3,4-trihydroxybenzoic acid molecules form stacks which extend down the *c*-axis giving weak π–π interactions [minimum ring centroid separation, 3.731 (3) Å], which is larger than that in the dihydrate form of the acid (Prior & Sharp, 2010) and in gallic acid pyridine monosolvate (Dong *et al.*, 2011), but comparable with those in natural flavonoids (Jiang *et al.*, 2002, 2009).

### Experimental

2,3,4-Trihydroxybenzoic acid (purity >98%) was purchased from the Sigma-Aldrich Company. Sample of 34 mg (0.2 mmol) was dissolved in methanol (1.5 ml) in a tube (4 ml) and sealed by parafilm. Light brown crystals were formed after three days at room temperature.

### Refinement

The C-bound H atoms were positioned geometrically and were included in the refinement in the riding-model approximation, with C—H = 0.96 Å (methyl C) and *U*_iso_(H) = 1.5*U*_eq_(C); 0.97 Å (methylene C) and *U*_iso_(H) = 1.2*U*_eq_(C); 0.93 Å (aryl H) and *U*_iso_(H)= 1.2*U*_eq_(C); O—H = 0.82 Å and *U*_iso_(H) = 1.5*U*_eq_(O).

### Crystal data

**Table:**

C_7_H_6_O_5_·0.25H_2_O	*F*(000) = 724
*M**_r_* = 174.62	*D*_x_ = 1.612 Mg m^−^^3^
Orthorhombic, *P*2_1_2_1_2	Mo *K*α radiation, λ = 0.71073 Å
Hall symbol: P 2ac 2ab	Cell parameters from 2552 reflections
*a* = 11.8364 (12) Å	θ = 2.1–50.0°
*b* = 32.598 (3) Å	µ = 0.14 mm^−^^1^
*c* = 3.7306 (4) Å	*T* = 291 K
*V* = 1439.4 (3) Å^3^	Prism, light brown
*Z* = 8	0.42 × 0.28 × 0.20 mm

### Data collection

**Table:**

Bruker SMART 1000 CCD diffractometer	2552 independent reflections
Radiation source: fine-focus sealed tube	2010 reflections with *I* > 2σ(*I*)
Graphite monochromator	*R*_int_ = 0.060
ω scan	θ_max_ = 25.0°, θ_min_ = 2.1°
Absorption correction: multi-scan (*SADABS*; Sheldrick, 2004)	*h* = −13→14
*T*_min_ = 0.874, *T*_max_ = 1.000	*k* = −38→34
7863 measured reflections	*l* = −4→4

### Refinement

**Table:**

Refinement on *F*^2^	Primary atom site location: structure-invariant direct methods
Least-squares matrix: full	Secondary atom site location: difference Fourier map
*R*[*F*^2^ > 2σ(*F*^2^)] = 0.036	Hydrogen site location: inferred from neighbouring sites
*wR*(*F*^2^) = 0.090	H-atom parameters constrained
*S* = 0.97	*w* = 1/[σ^2^(*F*_o_^2^) + (0.0443*P*)^2^] where *P* = (*F*_o_^2^ + 2*F*_c_^2^)/3
2552 reflections	(Δ/σ)_max_ < 0.001
222 parameters	Δρ_max_ = 0.17 e Å^−^^3^
0 restraints	Δρ_min_ = −0.15 e Å^−^^3^

### Special details

**Table:**

Geometry. All esds (except the esd in the dihedral angle between two l.s. planes) are estimated using the full covariance matrix. The cell esds are taken into account individually in the estimation of esds in distances, angles and torsion angles; correlations between esds in cell parameters are only used when they are defined by crystal symmetry. An approximate (isotropic) treatment of cell esds is used for estimating esds involving l.s. planes.
Refinement. Refinement of F^2^ against ALL reflections. The weighted R-factor wR and goodness of fit S are based on F^2^, conventional R-factors R are based on F, with F set to zero for negative F^2^. The threshold expression of F^2^ > 2sigma(F^2^) is used only for calculating R-factors(gt) etc. and is not relevant to the choice of reflections for refinement. R-factors based on F^2^ are statistically about twice as large as those based on F, and R- factors based on ALL data will be even larger.

### Fractional atomic coordinates and isotropic or equivalent isotropic displacement parameters (Å^2^)

**Table:**

	*x*	*y*	*z*	*U*_iso_*/*U*_eq_	
O1W	0.5000	0.0000	−0.1102 (7)	0.0395 (6)	
H1WA	0.5263	0.0173	0.0258	0.059*	
O1	0.65345 (12)	0.12870 (4)	0.2193 (5)	0.0334 (4)	
H1A	0.6637	0.1534	0.1900	0.040*	
O2	0.65656 (13)	0.04688 (4)	0.3549 (4)	0.0323 (4)	
H2A	0.6145	0.0640	0.4444	0.039*	
O3	0.84576 (14)	0.00203 (5)	0.2010 (5)	0.0445 (5)	
H3A	0.7859	−0.0048	0.2940	0.053*	
O4	0.75826 (14)	0.19200 (4)	−0.0346 (6)	0.0429 (5)	
O5	0.93058 (15)	0.18491 (4)	−0.2704 (6)	0.0481 (5)	
H5B	0.9243	0.2098	−0.2919	0.058*	
C1	0.84467 (19)	0.12653 (6)	−0.0250 (7)	0.0273 (5)	
C2	0.74982 (19)	0.10787 (6)	0.1337 (6)	0.0245 (5)	
C3	0.74932 (18)	0.06626 (6)	0.2074 (6)	0.0254 (5)	
C4	0.84311 (19)	0.04271 (6)	0.1272 (7)	0.0295 (5)	
C5	0.9374 (2)	0.06046 (7)	−0.0324 (7)	0.0330 (6)	
H5A	0.9998	0.0444	−0.0891	0.040*	
C6	0.9383 (2)	0.10180 (7)	−0.1064 (7)	0.0313 (6)	
H6A	1.0018	0.1135	−0.2117	0.038*	
C7	0.8406 (2)	0.17017 (7)	−0.1079 (7)	0.0310 (6)	
O1'	0.49399 (13)	0.17376 (4)	−0.2824 (5)	0.0359 (4)	
H1'A	0.4856	0.1982	−0.3270	0.043*	
O2'	0.49999 (12)	0.09083 (4)	−0.2151 (5)	0.0308 (4)	
H2'A	0.5380	0.1075	−0.1033	0.037*	
O3'	0.32290 (14)	0.04439 (4)	−0.4474 (5)	0.0397 (5)	
H3'A	0.3817	0.0378	−0.3464	0.048*	
O4'	0.39072 (15)	0.23703 (5)	−0.5434 (6)	0.0446 (5)	
O5'	0.22055 (15)	0.23003 (5)	−0.7935 (6)	0.0506 (5)	
H5'B	0.2294	0.2547	−0.8259	0.061*	
C1'	0.3088 (2)	0.17066 (7)	−0.5659 (7)	0.0293 (6)	
C2'	0.40325 (19)	0.15242 (6)	−0.4052 (6)	0.0271 (6)	
C3'	0.40863 (19)	0.11019 (6)	−0.3646 (6)	0.0267 (5)	
C4'	0.31874 (19)	0.08593 (6)	−0.4795 (7)	0.0281 (6)	
C5'	0.22322 (19)	0.10389 (7)	−0.6322 (7)	0.0328 (6)	
H5'A	0.1630	0.0876	−0.7056	0.039*	
C6'	0.2185 (2)	0.14569 (7)	−0.6737 (7)	0.0336 (6)	
H6'A	0.1546	0.1576	−0.7746	0.040*	
C7'	0.3098 (2)	0.21492 (7)	−0.6310 (7)	0.0349 (6)	

### Atomic displacement parameters (Å^2^)

**Table:**

	*U*^11^	*U*^22^	*U*^33^	*U*^12^	*U*^13^	*U*^23^
O1W	0.0469 (15)	0.0229 (11)	0.0486 (16)	−0.0056 (10)	0.000	0.000
O1	0.0299 (9)	0.0177 (7)	0.0527 (11)	0.0023 (6)	0.0057 (9)	0.0020 (8)
O2	0.0280 (9)	0.0212 (8)	0.0475 (11)	0.0003 (6)	0.0064 (8)	0.0020 (8)
O3	0.0386 (10)	0.0215 (8)	0.0734 (13)	0.0038 (7)	0.0121 (10)	0.0077 (10)
O4	0.0398 (11)	0.0244 (8)	0.0644 (13)	−0.0006 (8)	0.0084 (10)	0.0075 (9)
O5	0.0463 (11)	0.0268 (9)	0.0713 (14)	−0.0031 (8)	0.0157 (11)	0.0094 (10)
C1	0.0298 (13)	0.0244 (11)	0.0278 (13)	−0.0029 (10)	−0.0040 (11)	0.0009 (11)
C2	0.0265 (12)	0.0208 (11)	0.0261 (13)	0.0014 (9)	−0.0058 (11)	−0.0024 (10)
C3	0.0285 (13)	0.0211 (11)	0.0268 (12)	−0.0056 (9)	−0.0017 (11)	0.0004 (10)
C4	0.0334 (14)	0.0193 (11)	0.0359 (14)	0.0014 (10)	−0.0033 (12)	0.0009 (11)
C5	0.0278 (14)	0.0303 (13)	0.0409 (15)	0.0041 (10)	0.0006 (12)	−0.0034 (12)
C6	0.0257 (13)	0.0324 (13)	0.0360 (15)	−0.0042 (10)	−0.0001 (12)	0.0006 (11)
C7	0.0315 (14)	0.0255 (12)	0.0361 (14)	−0.0068 (11)	−0.0040 (12)	0.0019 (11)
O1'	0.0346 (9)	0.0185 (7)	0.0546 (11)	−0.0032 (6)	−0.0058 (10)	0.0011 (8)
O2'	0.0294 (9)	0.0191 (7)	0.0440 (10)	0.0016 (6)	−0.0038 (9)	0.0011 (7)
O3'	0.0408 (11)	0.0207 (8)	0.0575 (12)	−0.0029 (7)	−0.0115 (9)	−0.0004 (9)
O4'	0.0476 (12)	0.0208 (8)	0.0655 (14)	0.0004 (8)	−0.0142 (10)	0.0057 (9)
O5'	0.0482 (11)	0.0279 (9)	0.0758 (14)	0.0047 (8)	−0.0172 (12)	0.0138 (10)
C1'	0.0340 (14)	0.0227 (12)	0.0312 (13)	0.0031 (10)	0.0040 (11)	−0.0003 (11)
C2'	0.0295 (13)	0.0221 (12)	0.0297 (14)	−0.0024 (10)	0.0035 (12)	−0.0021 (10)
C3'	0.0280 (13)	0.0231 (11)	0.0290 (13)	0.0037 (10)	0.0045 (11)	0.0009 (10)
C4'	0.0334 (14)	0.0199 (11)	0.0311 (14)	0.0005 (10)	0.0052 (11)	−0.0007 (11)
C5'	0.0297 (14)	0.0299 (12)	0.0388 (15)	−0.0033 (10)	−0.0005 (12)	−0.0019 (12)
C6'	0.0342 (14)	0.0326 (12)	0.0339 (15)	0.0051 (10)	−0.0039 (12)	0.0022 (12)
C7'	0.0399 (16)	0.0271 (13)	0.0376 (15)	0.0071 (11)	0.0020 (12)	0.0021 (12)

### Geometric parameters (Å, º)

**Table:**

O1W—H1WA	0.8200	O1'—C2'	1.359 (3)
O1—C2	1.365 (3)	O1'—H1'A	0.8200
O1—H1A	0.8200	O2'—C3'	1.371 (3)
O2—C3	1.381 (3)	O2'—H2'A	0.8200
O2—H2A	0.8200	O3'—C4'	1.360 (2)
O3—C4	1.355 (2)	O3'—H3'A	0.8200
O3—H3A	0.8200	O4'—C7'	1.243 (3)
O4—C7	1.237 (3)	O5'—C7'	1.314 (3)
O5—C7	1.317 (3)	O5'—H5'B	0.8200
O5—H5B	0.8200	C1'—C2'	1.401 (3)
C1—C6	1.404 (3)	C1'—C6'	1.403 (3)
C1—C2	1.407 (3)	C1'—C7'	1.463 (3)
C1—C7	1.457 (3)	C2'—C3'	1.386 (3)
C2—C3	1.384 (3)	C3'—C4'	1.393 (3)
C3—C4	1.382 (3)	C4'—C5'	1.395 (3)
C4—C5	1.391 (3)	C5'—C6'	1.373 (3)
C5—C6	1.376 (3)	C5'—H5'A	0.9300
C5—H5A	0.9300	C6'—H6'A	0.9300
C6—H6A	0.9300		
			
C2—O1—H1A	109.5	C2'—O1'—H1'A	109.5
C3—O2—H2A	109.5	C3'—O2'—H2'A	109.5
C4—O3—H3A	109.5	C4'—O3'—H3'A	109.5
C7—O5—H5B	109.5	C7'—O5'—H5'B	109.5
C6—C1—C2	118.2 (2)	C2'—C1'—C6'	119.0 (2)
C6—C1—C7	122.8 (2)	C2'—C1'—C7'	118.9 (2)
C2—C1—C7	119.0 (2)	C6'—C1'—C7'	122.1 (2)
O1—C2—C3	115.9 (2)	O1'—C2'—C3'	115.8 (2)
O1—C2—C1	123.35 (18)	O1'—C2'—C1'	123.89 (18)
C3—C2—C1	120.7 (2)	C3'—C2'—C1'	120.3 (2)
O2—C3—C4	118.08 (17)	O2'—C3'—C2'	122.5 (2)
O2—C3—C2	122.06 (19)	O2'—C3'—C4'	117.79 (18)
C4—C3—C2	119.9 (2)	C2'—C3'—C4'	119.7 (2)
O3—C4—C3	121.2 (2)	O3'—C4'—C3'	120.7 (2)
O3—C4—C5	118.4 (2)	O3'—C4'—C5'	118.9 (2)
C3—C4—C5	120.4 (2)	C3'—C4'—C5'	120.4 (2)
C6—C5—C4	119.9 (2)	C6'—C5'—C4'	119.7 (2)
C6—C5—H5A	120.0	C6'—C5'—H5'A	120.1
C4—C5—H5A	120.0	C4'—C5'—H5'A	120.1
C5—C6—C1	120.9 (2)	C5'—C6'—C1'	120.8 (2)
C5—C6—H6A	119.6	C5'—C6'—H6'A	119.6
C1—C6—H6A	119.6	C1'—C6'—H6'A	119.6
O4—C7—O5	122.0 (2)	O4'—C7'—O5'	121.6 (2)
O4—C7—C1	122.7 (2)	O4'—C7'—C1'	122.3 (2)
O5—C7—C1	115.3 (2)	O5'—C7'—C1'	116.1 (2)
			
C6—C1—C2—O1	−178.9 (2)	C6'—C1'—C2'—O1'	−178.2 (2)
C7—C1—C2—O1	−0.8 (3)	C7'—C1'—C2'—O1'	4.6 (4)
C6—C1—C2—C3	0.1 (3)	C6'—C1'—C2'—C3'	2.1 (4)
C7—C1—C2—C3	178.2 (2)	C7'—C1'—C2'—C3'	−175.1 (2)
O1—C2—C3—O2	0.7 (3)	O1'—C2'—C3'—O2'	−0.9 (3)
C1—C2—C3—O2	−178.4 (2)	C1'—C2'—C3'—O2'	178.8 (2)
O1—C2—C3—C4	179.6 (2)	O1'—C2'—C3'—C4'	179.3 (2)
C1—C2—C3—C4	0.5 (3)	C1'—C2'—C3'—C4'	−1.0 (4)
O2—C3—C4—O3	−2.0 (3)	O2'—C3'—C4'—O3'	−0.8 (3)
C2—C3—C4—O3	179.0 (2)	C2'—C3'—C4'—O3'	179.0 (2)
O2—C3—C4—C5	177.9 (2)	O2'—C3'—C4'—C5'	179.7 (2)
C2—C3—C4—C5	−1.0 (4)	C2'—C3'—C4'—C5'	−0.5 (4)
O3—C4—C5—C6	−179.1 (2)	O3'—C4'—C5'—C6'	−178.6 (2)
C3—C4—C5—C6	1.0 (4)	C3'—C4'—C5'—C6'	0.9 (4)
C4—C5—C6—C1	−0.4 (4)	C4'—C5'—C6'—C1'	0.3 (4)
C2—C1—C6—C5	−0.1 (4)	C2'—C1'—C6'—C5'	−1.8 (4)
C7—C1—C6—C5	−178.2 (2)	C7'—C1'—C6'—C5'	175.3 (2)
C6—C1—C7—O4	−179.6 (2)	C2'—C1'—C7'—O4'	−1.2 (4)
C2—C1—C7—O4	2.3 (4)	C6'—C1'—C7'—O4'	−178.3 (2)
C6—C1—C7—O5	1.0 (3)	C2'—C1'—C7'—O5'	177.7 (2)
C2—C1—C7—O5	−177.1 (2)	C6'—C1'—C7'—O5'	0.6 (4)

### Hydrogen-bond geometry (Å, º)

**Table:**

*D*—H···*A*	*D*—H	H···*A*	*D*···*A*	*D*—H···*A*
O1*W*—H1*WA*···O2	0.82	2.19	2.963 (2)	156
O1*W*—H1*WA*···O2′	0.82	2.58	2.987 (3)	112
O1—H1*A*···O4	0.82	1.88	2.587 (3)	143
O2—H2*A*···O2′^i^	0.82	2.05	2.839 (4)	161
O2—H2*A*···O1	0.82	2.32	2.715 (2)	111
O3—H3*A*···O3′^ii^	0.82	2.06	2.828 (3)	155
O3—H3*A*···O2	0.82	2.29	2.735 (3)	115
O5—H5*B*···O4′^iii^	0.82	1.88	2.679 (4)	165
O1′—H1′*A*···O4′	0.82	1.87	2.588 (3)	145
O2′—H2′*A*···O1	0.82	1.95	2.729 (4)	159
O2′—H2′*A*···O1′	0.82	2.32	2.716 (2)	110
O3′—H3′*A*···O1*W*	0.82	2.06	2.841 (3)	158
O3′—H3′*A*···O2′	0.82	2.28	2.727 (4)	115
O5′—H5′*B*···O4^iv^	0.82	1.85	2.659 (4)	171

Symmetry codes: (i) *x*, *y*, *z*+1; (ii) −*x*+1, −*y*, *z*+1; (iii) *x*+1/2, −*y*+1/2, −*z*−1; (iv) *x*−1/2, −*y*+1/2, −*z*−1.
